# The Surgeon General

**Published:** 1864-02

**Authors:** 


*The Surgeon General.*—During the progress of the present sanguinary struggle, no department of our vast organization to crush the rebellion has been regarded by an intelligent and thoughtful people, with more anxiety than the medical; and its progress and augmenting efficiency have been watched by the medical profession throughout the country with a nervous interest. The necessity to the army of a well organized medical staff was fully appreciated long ago. Says Xenophon, "The great Cyrus never failed to take a certain number of excellent physicians along with him in the army, rewarding them very liberally, and treating them with particular regard. And in this he only followed a custom that had been anciently established by their generals." From the earliest times medical men have not only rendered invaluable service to the army as physicians, but they have frequently signalized themselves on the field as military leaders. "Podalirius and Machaon both commanded troops at the siege of Troy. They were no less excellent physicians than brave officers, and rendered as much service to the Greeks by their skill in medicine as they did by their courage and conduct in their military capacity." And a similar statement may be made of the representatives of the medical profession in every great military campaign from that time down to our own great conflict for the preservation of the country. And yet there is a feeling prevalent, not confined to the medical profession, that the medical element in our present war has not been fully appreciated, or treated with proper consideration. A large share of the malignity against the profession having its origin with those who would favor quackery, in some one or other of its many forms, has been directed against the head of the Medical

Department of the Army, Surgeon General Hammond. Malfeasance, with other charges, has been urged against that officer. Of the justness of this charge we know nothing, and express no opinion, and can only hope that the Surgeon General, in compliance with his own request for an investigation, may have an impartial trial.

We consider the question of his competency for the position as already answered,

1st. By what he has accomplished in rendering the medical organization efficient, the following brief extract from a London cotemporary expresses our views, and shows how a disinterested party regards him:

"Appointed by the President, in spite of the old routine custom, over the heads of many seniors, he came to his task full of vigor, in the prime of life, and capable of great physical endurance. With a bold hand he surrounded himself with trustworthy subordinates, displacing many whom he did not think equal to the crisis, and proceeded energetically with his work. Large armies had to be provided for, a system of military hospitals to be organized, the examining boards to be reconstructed, and an army medical school and museum to be founded. Well, in these vast and useful works he seems to have succeeded beyond all expectation, and the confidence of the public in the new system of medical organization has been warmly expressed, and yet by the last accounts we learn that he has been suspended from his office, and ordered to a distant service, a commission having been appointed to inquire into the condition and management of his office."

2d. By the fact that the department has been well supplied with medical stores and appliances, in every emergency, so far as human prescience could anticipate the demand.

3d. By the fact that our army, though raised in the higher latitudes and has been on duty in the malarious districts of the South, has not suffered from the prevalence of fatal epidemics, such as have decimated the armies of all nations, under similar circumstances. And we cheerfully append the Address of the U. S. Sanitary Commission to the President, as the testimony of men whose opinions should have weight:

### U. S. SANITARY COMMISSION.

*To his Excellency the President of the United States:*

SIR :—The United States Sanitary Commission authorized by the Government to act as a Commission of inquiry and Advice in respect to the sanitary interests of the national forces, have been for more than two years and a half close and careful students of the medical and hygienic affairs of the army. They ought to be, they are thought by the people of the United States to be, they claim to be, better acquainted with the working of the Medical Department, whose deficiencies, mistakes, and necessities, it is their solemn duty to discover and obviate, than any other responsible body of witnesses. Trusting in their discretion, zeal, and works, the people of the loyal States have made them their almoners, to the extent of seven millions of dollars' worth of sanitary stores, and a million of dollars in money. The disbursement of this immense charity has brought our agents into close and continual contact with the Medical Department, of whose steady and rapid improvement from the imperfect state in which we found it, to its present degree of surprising and gratifying efficiency, we are able to lend a most indisputable testimony. We attribute this immense improvement to the fact that for two years the Medical Department has been directed by Dr. W. A. HAMMOND, Surgeon General, a man known to all impartial and competent judges, as thoroughly scientific, highly endorsed, large-minded, and an energetic and controlling administrator. He was selected for his office, solely for fitness, and in our calm and deliberate judgment, his administration has more than justified all the high hope and expectations of those who recommended him for the place.

We hear from sources that do not permit us to doubt the fact, that cautious but systematic efforts are now making to remove Surgeon General HAMMOND from office. In the name of some millions of constituents, in the name of the homes of this country, whose solicitude, liberality, and watchfulness, we represent, we respectfully and conscientiously protest against the secret tribunal, and the indirect methods, by which the good fame of the Surgeon General has been already seriously, and we believe unjustly, aspersed. We protest, in our character of experts, a body whose business it has been made to inquire and advise on this very subject, that the removal of Dr. HAMMOND would be as serious a blow, at the lives, comfort, and efficiency of the army, as the enemy itself could inflict; that the science of the country, the humanity of its homes, and the army itself, would resent it as a cruel wrong and an alarming error; and we feel ourselves bound, in the interests of the soldiers in the field, and of those about to enter the service of the country, in the defence of our own principles and convictions, and in the name of the science, the charity, and the fair-mindedness of the nation, to beg that no further steps in this direction may be taken, without a full and fair trial of Surgeon General HAMMOND upon the charges alleged to have been secretly made against him.

Dec. 29, 1863.

Signed { H. W. BELLOWS, WM. H. VAN BUREN, WOLCOTT GIBBS, GEO. T. STRONG, C. R. AGNEW,

Standing Committee U. S. Sanitary Commission.

